# Behavioral and Psychological Symptoms of Dementia and Mortality Risk Among People With Cognitive Impairment: An 8-year Longitudinal Study From the NCGG-STORIES

**DOI:** 10.2188/jea.JE20230343

**Published:** 2024-11-05

**Authors:** Taiji Noguchi, Takeshi Nakagawa, Taiki Sugimoto, Ayane Komatsu, Yujiro Kuroda, Kazuaki Uchida, Rei Ono, Hidenori Arai, Takashi Sakurai, Tami Saito

**Affiliations:** 1 Department of Social Science, Research Institute, National Center for Geriatrics and Gerontology, Aichi, Japan; 2 Department of Behavioural Science and Health, Institute of Epidemiology and Health Care, University College London, London, United Kingdom; 3 Japan Society for the Promotion of Science, Tokyo, Japan; 4 Department of Medicine, University of Washington, Seattle, United States; 5 Department of Prevention and Care Science, Research Institute, National Center for Geriatrics and Gerontology, Aichi, Japan; 6 Center for Comprehensive Care and Research on Memory Disorders, Hospital, National Center for Geriatrics and Gerontology, Aichi, Japan; 7 Department of Physical Activity Research, National Institutes of Biomedical Innovation, Health and Nutrition, Osaka, Japan; 8 Department of Public Health, Graduate School of Health Sciences, Kobe University, Kobe, Japan; 9 National Center for Geriatrics and Gerontology, Aichi, Japan; 10 Research Institute, National Center for Geriatrics and Gerontology, Aichi, Japan; 11 Department of Cognition and Behavior Science, Nagoya University Graduate School of Medicine, Nagoya, Japan

**Keywords:** behavioral and psychological symptoms of dementia, BPSD, cognitive impairment, dementia, prognosis

## Abstract

**Background:**

Behavioral and psychological symptoms of dementia (BPSD) are common among people with dementia from the early stages and can appear even in mild cognitive impairment (MCI). However, the prognostic impact of BPSD is unclear. This study examined the association between BPSD and mortality among people with cognitive impairment.

**Methods:**

This longitudinal study involved 1,065 males and 1,681 females (mean age: 77.1 years for males and 78.6 years for females) with MCI or dementia diagnosis from the National Center for Geriatrics and Gerontology-Life Stories of People with Dementia (NCGG-STORIES), a single-center memory clinic-based cohort study in Japan that registered first-time outpatients from 2010–2018. Information about death was collected through a mail survey returned by participants or their close relatives, with an up to 8-year follow-up. BPSD was assessed using the Dementia Behavior Disturbance Scale (DBD) at baseline.

**Results:**

During the follow-up period, 229 (28.1%) male and 254 (15.1%) female deaths occurred. Cox proportional hazards regression analysis showed that higher DBD scores were significantly associated with increased mortality risk among males, but not females (compared with the lowest quartile score group, hazard ratios for the highest quartile score group were 1.59; 95% confidence interval, [CI] 1.11–2.29 for males and 1.06; 95% CI, 0.66–1.70 for females). Among the DBD items, lack of interest in daily living, excessive daytime sleep, and refusal to receive care had a higher mortality risk.

**Conclusion:**

The findings suggest a potential association between BPSD and poor prognosis among males with cognitive impairment.

## INTRODUCTION

Dementia, a progressive neurodegenerative disease characterized by cognitive decline, is a major healthcare challenge.^1^ The estimated number of people with dementia worldwide was 57.4 million in 2019 and is expected to increase to 152.8 million by 2050.^2^ Given the rising global disease burden due to dementia, understanding the disease, including its prognosis, and improving dementia care are important issues.

Most people with dementia experience progressive cognitive decline as well as non-cognitive symptoms, such as behavioral and psychological symptoms of dementia (BPSD).^3^^,^^4^ BPSD are defined as “symptoms of disturbed perception, thought content, mood or behavior that frequently occur in patients with dementia.”^5^ Specifically, BPSD include psychological symptoms, such as anxiety, depressive mood, hallucinations, and delusions, and behavioral symptoms, such as aggression, restlessness, agitation, wandering, culturally inappropriate behaviors, and sexual disinhibition.^4^^,^^5^ BPSD are more common in severe dementia,^6^ but are expressed at all stages of dementia, even the early stage, and are also observed in mild cognitive impairment (MCI).^4^^,^^7^^–^^9^ Because BPSD have a large social impact, such as increased caregiver burden^10^^,^^11^ and institutionalization risk,^12^^–^^14^ addressing BPSD from the early stage is critical in dementia care.

BPSD may be associated with poor prognosis, including early death, but the evidence is insufficient. Earlier previous research has indicated that behavioral disorders, such as wandering and aggression, and psychiatric symptoms, such as hallucinations and apathy, were associated with a higher mortality risk among people with dementia,^15^^–^^20^ although some results conflict.^21^^,^^22^ Subsequent studies have not found a mortality risk of BPSD.^23^^–^^25^ These included relatively small sample sizes and inadequate consideration of confounders, which may have led to inconsistencies among the results. Recently, a large study of nursing home patients with dementia reported that those with moderate or severe BPSD had a higher mortality risk, with hallucinations, agitation, apathy, and eating changes as contributors, re-emphasizing the adverse prognostic impact of BPSD.^26^ Nevertheless, evidence of the association including those with early-stage dementia and MCI still remains limited. Considering that BPSD appears from the early stages of the disease stage,^4^^,^^7^^–^^9^ addressing this research gap may assist in understanding the prognostic impact of BPSD. Furthermore, the frequency and types of BPSD and the survival time and the related factors differ between males and females among people with dementia.^27^^–^^34^ For instance, aggression and disinhibition, which is more pronounced in males,^28^ may interfere with seeking care and treatment, and negative symptoms, which are more common in females,^27^^,^^29^^,^^31^ may promote inactivity and decreased functioning; these sex specificity of BPSD can lead to sex differences in prognosis. However, little research has addressed the sex differences, which may account for the inconsistent evidence for the association between BPSD and mortality risk. Additionally, given the need to consider sex differences in dementia care,^35^ examining sex differences can help understand the sex-specific needs of people with dementia.

This study aimed to examine the association between BPSD and mortality among people with cognitive impairment. Particularly, we also included the participants with early-stage cognitive impairment, including MCI, and further examined the sex differences.

## METHODS

### Study participants

This single center-based longitudinal study used data from the National Center for Geriatrics and Gerontology-Life Stories of People with Dementia (NCGG-STORIES), the clinical records and prognostic data of patients who consulted the NCGG’s memory clinic in Japan.^36^^,^^37^ In total, 4,952 first-visit patients (aged 33–98 years) were registered from July 2010–September 2018 and followed up with mailed questionnaires from November 2018–January 2019. Consequently, 3,731 returned the questionnaires (response rate: 79.7%). Of them, 985 were excluded because they did not meet the clinical diagnosis criteria for dementia or MCI as defined by the National Institute on Aging-Alzheimer’s Association workgroups.^38^^,^^39^ Finally, 2,746 participants (aged 44–98 years), including 75 individuals with early-onset dementia, were included in the analysis. This study protocol was approved by the Ethics Committee of the National Center for Geriatrics and Gerontology (No. 1180-2). The purpose, nature, and potential risks of this study were fully explained to the participants or their close relatives, and written informed consent was provided before study participation.

### Mortality

Participants were followed for death information through a mailed questionnaire survey from November 2018–January 2019. The participants or close relatives responded with the participants’ death-related information, including survival status and year and month of death, when they received the questionnaire, and we calculated the days until death. A high accuracy of the death-related information from the questionnaires was reported in our previous study.^36^

### Behavioral and psychological symptoms of dementia

BPSD were assessed at the first visit using the Japanese version of the Dementia Behavior Disturbance Scale (DBD) completed by a close relative^40^^,^^41^; the scale has been verified for validity and reliability.^40^ The DBD comprises 28 items on non-cognitive behaviors often observed in people with cognitive impairment. Each item is rated on a Likert-type scale with five levels of frequency for the behavior observed in the previous week (0 = “never,” 1 = “rarely,” 2 = “some of the time,” 3 = “often,” and 4 = “most of the time”). The DBD is scored from 0–112 points (higher scores indicate more severe BPSD). The total score and item count distributions and summary statistics are shown in eTable 1 and eFigure 1, respectively. The total score was divided into quartiles by sex. Additionally, we used the counts of “often” or “most of the time” items of the 28-item DBD, and the total item counts were divided into 4 groups based on the distribution: “zero,” “one,” “two to three,” and “four or more.”

### Covariates

The covariates were obtained at the first visit and included age, living arrangement, education, economic status, body mass index (BMI), basic and instrumental activities of daily living (BADL and IADL, respectively), comorbidities, depressive symptoms, dementia types, antidementia and psychoactive drug use, and cognitive function. Age was categorized as <70, 70–74, 75–79, 80–84, and ≥85 years. Living arrangement was dichotomized as “living together” and “living alone.” Education was categorized as ≤9, 10–12, and ≥13 years. Economic status was categorized as “poor,” “normal,” and “affordable.” BMI was categorized as <18.5, 18.5–24.9, and ≥25.0 kg/m^2^. BADL assessed using the Barthel Index (BI) was categorized as 100, 85–99, 60–84, and ≤59 points.^42^ IADL was assessed using the Lawton Index, including 5 items (males) and 8 items (females); those with difficulty on ≥1 item were labeled “with difficulty,” while others as “without difficulty.”^43^ Comorbidities were assessed using self-reported for cancer, heart disease, stroke, hypertension, dyslipidemia, diabetes, kidney disease, liver disease, and lung disease (“yes” or “no”). Although depressive symptoms are often identified as one feature of BPSD, we included depressive symptoms as a covariate because the DBD does not explicitly evaluate psychological symptoms, including depressive symptoms; it was assessed using the Geriatrics Depression Scale 15-item, with a score of ≥5 points defined as “depressed,” others as “not depressed.”^44^ Dementia types diagnosed by medical doctors according to the international criteria^38^^,^^39^ were classified as MCI, Alzheimer’s disease (AD), dementia with Lewy bodies (DLB), frontotemporal lobar degeneration (FLTD), and vascular dementia (VAD). Drug use was referred from medical records; antidementia drug was categorized as none, acetylcholine inhibitors (AChE inhibitors), NMDA receptor antagonists (NMDAR antagonists), and both; psychoactive drug was dichotomized as “yes” or “no.” Cognitive function assessed using the Mini-Mental State Examination (MMSE) was categorized as 30–26, 25–21, 20–11, and 10–0 points.^45^

### Statistical analyses

All statistical analyses were conducted by sex, considering that the survival time and the related factors differ by sex among people with dementia.^33^ First, the incidence rate of death by participants’ characteristics was calculated. Second, DBD scores and frequency of each item (‘often’ or ‘most of the time’) were summarized. Third, to examine the association between BPSD and mortality, we performed survival analysis with the Cox proportional hazards model and obtained the hazard ratios (HRs) and 95% confidential intervals (CIs) for mortality, considering models that introduced either DBD scores or item counts (the proportional hazard assumption was assessed a priori; eFigure 2). To address potential bias resulting from missing information, the multiple imputation approach under the missing at random assumption was applied. We generated 20 imputed datasets using the multiple imputation by chained equations procedure and pooled the results using the standard Rubin’s rule.^46^ Additionally, we conducted a complete case analysis.

Sensitivity analyses were conducted excluding events occurring within 6, 12, 18, or 24 months of follow-up, respectively, to address the reverse causality of BPSD and death. Additionally, we performed the analyses restricted to participants with AD and excluded those with each dementia type to eliminate the dementia type effects.

Stratified analyses were performed by BADL (BI <100 [difficulty] or 100 points [not difficulty]) and cognitive function (MMSE <21 [moderately/severely impaired] or ≥21 points [mildly impaired]) to address the proxy influence of ADL or cognitive dysfunction on BPSD and prognosis. Interaction tests were also conducted.

We analyzed the association of 28 DBD items individually with mortality. The analytical model that entered individual DBD items with a frequency of ≥5% for each sex was performed.

The significance level was set at <0.05. All statistical analyses used R Version 4.2.2 for Windows (R Foundation for Statistical Computing, Vienna, Austria).

## RESULTS

Data from 1,065 males and 1,681 females were analyzed. The participants’ mean follow-up days were 1,389.3 (range, 50–2,990) for males and 1,589.5 (range, 50–3,032) for females. During the follow-up, 299 (28.1%) males and 254 (15.1%) females died.

Table 1 presents the baseline characteristics. The mean age was 77.1 (standard deviation [SD], 7.3) years for males and 78.6 (SD, 7.2) years for females. The incidence rate of mortality across sexes was higher in those who were older, lived together, less educated, poorer economic status, lower BMI, had ADL impairment, had cancer, heart disease, diabetes, liver disease, and pulmonary disease, depressed, had dementia diagnosis, used antidementia drugs, and had lower cognitive function. Regarding the DBD, both males and females with higher scores and more item counts had a higher mortality incidence rate.

**Table 1.** Baseline characteristics of the study participants

		Males (*n* = 1,065)		Females (*n* = 1,681)	
	
		*n* (%)	Incidence rate (per 1,000 person-years)	*n* (%)	Incidence rate (per 1,000 person-years)
Age, years	<70	152 (14.3)	25.8	182 (10.8)	7.0
	70–74	195 (18.3)	47.4	250 (14.9)	25.2
	75–79	307 (28.8)	67.8	403 (24.0)	25.2
	80–84	250 (23.5)	96.7	506 (30.1)	38.8
	≥85	161 (15.1)	156.1	340 (20.2)	66.9
Living arrangement	Living together	645 (60.6)	78.6	1,021 (60.7)	38.8
	Living alone	407 (38.2)	58.0	646 (38.4)	24.6
	Missing	13 (1.2)	59.1	14 (0.8)	13.6
Education, years	<9	402 (37.7)	85.5	919 (54.7)	37.3
	10–12	361 (33.9)	78.5	597 (35.5)	33.5
	≥13	292 (27.4)	49.9	138 (8.2)	19.0
	Missing	10 (0.9)	155.6	27 (1.6)	57.8
Economic status	Affordable	182 (17.1)	69.7	371 (22.1)	32.9
	Normal	826 (77.6)	74.4	1,169 (69.5)	34.8
	Poor	44 (4.1)	90.1	124 (7.4)	41.0
	Missing	13 (1.2)	50.9	17 (1.0)	22.6
BMI, kg/m^2^	<18.5	83 (7.8)	162.6	273 (16.2)	39.7
	18.5–24.9	768 (72.1)	73.1	1,116 (66.4)	35.4
	≥25.0	210 (19.7)	48.4	289 (17.2)	26.8
	Missing	4 (0.4)	113.6	3 (0.2)	58.8
Barthel Index, points	100	751 (70.5)	51.4	1,168 (69.5)	25.0
	85–99	192 (18.0)	119.3	319 (19.0)	52.1
	60–84	77 (7.2)	168.8	132 (7.9)	61.8
	<60	36 (3.4)	188.7	58 (3.5)	84.8
	Missing	9 (0.8)	31.6	4 (0.2)	41.3
IADL performance	Without difficulty	314 (29.5)	33.1	483 (28.7)	14.2
	With difficulty	740 (69.5)	93.0	1,193 (71.0)	43.1
	Missing	11 (1.0)	21.2	5 (0.3)	33.4
Cancer	No	932 (87.5)	72.9	1,561 (92.9)	33.9
	Yes	124 (11.6)	84.7	106 (6.3)	49.2
	Missing	9 (0.8)	48.8	14 (0.8)	31.7
Heart disease	No	851 (79.9)	70.5	1,483 (88.2)	31.1
	Yes	205 (19.2)	89.8	184 (10.9)	66.5
	Missing	9 (0.8)	48.8	14 (0.8)	31.7
Stroke	No	951 (89.3)	73.6	1,592 (94.7)	35.5
	Yes	105 (9.9)	79.9	75 (4.5)	20.4
	Missing	9 (0.8)	48.8	14 (0.8)	31.7
Hypertension	No	605 (56.8)	78.9	855 (50.9)	30.1
	Yes	451 (42.3)	67.7	812 (48.3)	39.8
	Missing	9 (0.8)	48.8	14 (0.8)	31.7
Dyslipidemia	No	911 (85.5)	78.8	1,336 (79.5)	35.8
	Yes	145 (13.6)	48.3	331 (19.7)	30.5
	Missing	9 (0.8)	48.8	14 (0.8)	31.7
Diabetes	No	861 (80.8)	70.7	1,440 (85.7)	32.1
	Yes	195 (18.3)	90.6	227 (13.5)	52.3
	Missing	9 (0.8)	48.8	14 (0.8)	31.7
Kidney disease	No	1,017 (95.5)	74.1	1,635 (97.3)	34.2
	Yes	39 (3.7)	76.1	32 (1.9)	64.5
	Missing	9 (0.8)	48.8	14 (0.8)	31.7
Liver disease	No	1,019 (95.7)	73.7	1,633 (97.1)	34.2
	Yes	37 (3.5)	87.4	34 (2.0)	60.0
	Missing	9 (0.8)	48.8	14 (0.8)	31.7
Pulmonary disease	No	983 (92.3)	71.4	1,610 (95.8)	34.0
	Yes	73 (6.9)	116.9	57 (3.4)	58.3
	Missing	9 (0.8)	48.8	14 (0.8)	31.7
Depressive symptoms	Not depressed	670 (62.9)	62.8	1,004 (59.7)	29.3
	Depressed	370 (34.7)	89.6	630 (37.5)	38.5
	Missing	25 (2.3)	120.4	47 (2.8)	86.1
Dementia diagnosis	MCI	396 (37.2)	35.9	469 (27.9)	15.9
	AD	520 (48.8)	87.5	1,067 (63.5)	37.6
	DLB	74 (6.9)	154.5	99 (5.9)	71.6
	FTLD	24 (2.3)	116.8	21 (1.2)	57.3
	VAD	51 (4.8)	95.4	25 (1.5)	70.3
Antidementia drug use	None	888 (83.4)	67.9	1,360 (80.9)	35.2
	AChE inhibitors	115 (10.8)	113.3	217 (12.9)	35.6
	NMDAR antagonists	19 (1.8)	120.7	18 (1.1)	46.9
	Both	17 (1.6)	50.7	25 (1.5)	29.3
	Missing	26 (2.4)	61.1	61 (3.6)	26.1
Psychotropic drug use	No	924 (86.8)	75.4	1,383 (82.3)	36.0
	Yes	141 (13.2)	60.8	298 (17.7)	26.3
MMSE score, points	26–30	241 (22.6)	30.8	278 (16.5)	11.6
	21–25	396 (37.2)	54.7	561 (33.4)	27.8
	11–20	378 (35.5)	117.7	755 (44.9)	44.3
	0–10	47 (4.4)	158.1	81 (4.8)	67.3
	Missing	3 (0.3)	0.0	6 (0.4)	127.6
DBD total score	Q1 (lowest)	303 (28.5)	46.0	427 (25.4)	17.6
	Q2	247 (23.2)	51.2	387 (23.0)	30.1
	Q3	240 (22.5)	77.0	413 (24.6)	38.8
	Q4 (highest)	235 (22.1)	134.3	387 (23.0)	49.9
	Missing	40 (3.8)	80.7	67 (4.0)	56.2
DBD item counts	None	342 (32.1)	45.5	438 (26.1)	24.8
	One	245 (23.0)	53.2	367 (21.8)	26.1
	Two to three	256 (25.0)	90.3	519 (32.2)	35.2
	Four or more	182 (17.8)	137.7	290 (18.0)	54.2
	Missing	40 (3.8)	80.7	67 (4.0)	56.2

AChE, acetylcholine; AD, Alzheimer’s dementia; BMI, Body Mass Index; DBD, Dementia Behavior Disturbance Scale; DLB, dementia with Lewy bodies; FTLD, frontotemporal lobar degeneration; IADL, instrumental activity of daily living; MCI, mild cognitive impairment; MMSE, Mini-Mental State Examination; NMDAR, N-methyl-D-aspartate receptor; VAD, vascular dementia.

DBD scores: males, 0–7 points (Q1), 8–12 points (Q2), 13–20 points (Q3), and 21–70 points (Q4); females, 0–7 points (Q1), 8–12 points (Q2), 13–20 points (Q3), and 21–83 points (Q4).

Table 2 shows the descriptive statistics for the DBD by sex. The mean of the total scores were 14.7 (SD, 11.5) and 15.2 (SD, 11.6) and that of total item counts were 1.9 (SD, 2.4) and 2.1 (SD, 2.4) among males and females, respectively. For both sexes, #1 “Asks same question repeatedly” had the highest frequency: 42.2% (males) and 57.8% (females). Items with a frequency of ≥5% were: 12 for males (#1, 2, 3, 4, 5, 6, 8, 9, 12, 13, 19, and 20) and 10 for females (#1, 2, 3, 5, 6, 8, 9, 12, 13, and 19).

**Table 2.** DBD scores and item frequency

	Males (*n* = 1,065)	Females (*n* = 1,681)
	
	*n* (%)	*n* (%)
Total DBD score, mean (SD)	14.8 (11.5)	15.2 (11.6)
Total DBD item count, mean (SD)	1.9 (2.4)	2.1 (2.4)

Frequency of DBD 28 items^a^		
#1 Asks same question repeatedly	445 (42.2)	966 (57.8)
#2 Loses, misplaces, or hides things	316 (30.1)	779 (46.8)
#3 Lack of interest in daily activities	222 (21.2)	305 (18.4)
#4 Wakes up at night for no obvious reason	65 (6.2)	67 (4.0)
#5 Makes unwarranted accusations	58 (5.5)	87 (5.2)
#6 Sleeps excessively during the day	215 (20.4)	213 (12.8)
#7 Paces up and down	47 (4.5)	66 (3.9)
#8 Repeats the same action over and over	67 (6.4)	97 (5.8)
#9 Is verbally abusive or curses	64 (6.1)	83 (5.0)
#10 Dresses inappropriately	48 (4.6)	69 (4.1)
#11 Cries or laughs inappropriately	12 (1.1)	35 (2.1)
#12 Refuses to be helped with personal care	66 (6.3)	89 (5.3)
#13 Hoards things for no obvious reason	78 (7.4)	231 (13.8)
#14 Moves arms or legs in a restless or agitated way	24 (2.3)	24 (1.4)
#15 Empties drawers or closets	25 (2.4)	66 (4.0)
#16 Wanders in the house at night	19 (1.8)	20 (1.2)
#17 Gets lost outside	24 (2.3)	22 (1.3)
#18 Refuses to eat	6 (0.6)	15 (0.9)
#19 Overeats	71 (6.8)	93 (5.6)
#20 Is incontinent of urine	65 (6.2)	82 (4.9)
#21 Wanders aimlessly outside or in the house during the day	28 (2.7)	36 (2.2)
#22 Makes physical attacks (eg, hits, bites, scratches, kicks, or spits)	7 (0.7)	7 (0.4)
#23 Screams for no reason	12 (1.1)	8 (0.5)
#24 Makes inappropriate sexual advances	5 (0.5)	1 (0.1)
#25 Exposes private body parts	5 (0.5)	2 (0.1)
#26 Destroys property or clothing	2 (0.2)	5 (0.3)
#27 Is incontinent of stool	13 (1.2)	28 (1.7)
#28 Throws food	2 (0.2)	1 (0.1)

DBD, Dementia Behavior Disturbance Scale. SD, standard deviation.

^a^Frequency of “often” or “most of the time.”

Table 3 shows the association between DBD and mortality, based on the Cox proportional hazards regression analysis. Among males, higher DBD scores were associated with increased mortality risk, after adjusting for covariates (model 2): HR of morality, compared with the DBD score in Q1, for Q2 was 1.06 (95% CI, 0.71–1.58), for Q3 was 1.28 (95% CI, 0.89–1.86), and for Q4 was 1.59 (95% CI, 1.11–2.29) (*P for trend* = 0.008, *P for trend per one point* = 0.006). Similarly, higher DBD item counts were associated with an increased mortality risk (model 2): HR of mortality, compared with the DBD item counts in zero, for one was 0.98 (95% CI, 0.67–1.44), for two to three was 1.48 (95% CI, 1.04–2.09), and for four or more was 1.55 (95% CI, 1.06–2.27) (*P for trend* = 0.005, *P for trend per one item* = 0.002). However, among females, DBD scores and item counts were not associated with mortality risk (model 2): HR of mortality, compared with the DBD score in Q1, for Q2 was 1.24 (95% CI, 0.78–1.96), for Q3 was 1.33 (95% CI, 0.85–2.08), and for Q4 was 1.06 (95% CI, 0.66–1.70) (*P for trend* = 0.960, *P for trend per one point* = 0.589); HR of mortality, compared with the DBD item counts in zero, for one was 0.86 (95% CI, 0.56–1.31), for two to three was 0.89 (95% CI, 0.61–1.31), and for four or more was 1.02 (95% CI, 0.67–1.55) (*P for trend* = 0.862, *P for trend per one item* = 0.768). Complete case analysis showed similar trends for the association between DBD and mortality risk in males (eTable 2).

**Table 3.** Association between DBD and mortality, based on multivariable Cox proportional hazards regression analysis

	Males				Females			
	
	Model 1 (crude model)		Model 2 (adjusted model)^a^		Model 1 (crude model)		Model 2 (adjusted model)^a^	
			
	HR (95% CI)	*P*-value	HR (95% CI)	*P*-value	HR (95% CI)	*P*-value	HR (95% CI)	*P*-value
DBD total score								
Q1 (lowest)	1.00		1.00		1.00		1.00	
Q2	1.09 (0.74–1.61)	0.671	1.06 (0.71–1.58)	0.768	1.61 (1.03–2.51)	0.037	1.24 (0.78–1.96)	0.367
Q3	1.65 (1.15–2.35)	0.006	1.28 (0.89–1.86)	0.188	2.07 (1.36–3.13)	0.001	1.33 (0.85–2.08)	0.212
Q4 (highest)	2.97 (2.14–4.12)	<0.001	1.59 (1.11–2.29)	0.013	2.63 (1.75–3.95)	<0.001	1.06 (0.66–1.70)	0.819
	*P* for trend < 0.001		*P* for trend = 0.008		*P* for trend < 0.001		*P* for trend = 0.960	
	*P* for trend per one point < 0.001		*P* for trend per one point = 0.006		*P* for trend per one point < 0.001		*P* for trend per one point = 0.589	

DBD item counts								
None	1.00		1.00		1.00		1.00	
One	1.15 (0.79–1.66)	0.467	0.98 (0.67–1.44)	0.930	1.15 (0.76–1.73)	0.504	0.86 (0.56–1.31)	0.473
Two to three	2.07 (1.49–2.87)	<0.001	1.48 (1.04–2.09)	0.030	1.40 (0.98–2.00)	0.063	0.89 (0.61–1.31)	0.568
Four or more	3.19 (2.32–4.40)	<0.001	1.55 (1.06–2.27)	0.023	2.19 (1.52–3.15)	<0.001	1.02 (0.67–1.55)	0.941
	*P* for trend < 0.001		*P* for trend = 0.005		*P* for trend < 0.001		*P* for trend = 0.862	
	*P* for trend per one item < 0.001		*P* for trend per one item = 0.002		*P* for trend per one item < 0.001		*P* for trend per one item = 0.768	

CI, confidence interval; DBD, Dementia Behavior Disturbance Scale; HR, hazard ratio.

^a^Adjusted for age, living arrangement, education, economic status, Body Mass Index, basic activities of daily living (ADL), instrumental ADL, comorbidities, depressive symptoms, dementia types, antidementia drug use, psychotropic drug use, and cognitive function.

Missing data were imputed using a multiple imputation approach.

The sensitivity analyses excluding participants with events within 6–24 months from baseline indicated broadly similar tendencies in the results of the association between DBD and mortality risk in males (eTable 3). Additionally, analyses restricted to those with AD or excluding those with each dementia type yielded similar results to the main analysis (eTable 4).

Table 4 shows the results of stratified analyses by BADL or cognitive function. The results indicated a similar direction for the association between DBD and mortality risk in males with and without BADL disabilities or cognitive impairment. However, the association was more marked in those with BADL disabilities, although the interaction was not found to be statistically significant (eTable 5).

**Table 4.** Stratified analyses by BADL or cognitive function on association between DBD and mortality, based on multivariable Cox proportional hazards regression analysis

	BADL				Cognitive function			
	
	With difficulty in BADL^a^		Without difficulty in BADL^a^		With moderately/severely impaired cognitive function^b^		With mildly impaired cognitive function^b^	
			
	HR (95% CI)	*P*-value	HR (95% CI)	*P*-value	HR (95% CI)	*P*-value	HR (95% CI)	*P*-value
***Males***								
DBD total score								
Q1 (lowest)	1.00		1.00		1.00		1.00	
Q2	1.52 (0.57–4.05)	0.404	0.97 (0.60–1.57)	0.905	1.13 (0.61–2.08)	0.699	0.93 (0.53–1.63)	0.797
Q3	2.12 (0.87–5.14)	0.101	1.02 (0.63–1.64)	0.949	1.17 (0.68–2.01)	0.570	1.40 (0.81–2.43)	0.237
Q4 (highest)	2.45 (1.05–5.71)	0.040	1.66 (1.00–2.76)	0.052	1.58 (0.94–2.67)	0.086	1.38 (0.74–2.54)	0.312
	*P* for trend = 0.019		*P* for trend = 0.106		*P* for trend = 0.066		*P* for trend = 0.163	
	*P* for trend per one point = 0.034		*P* for trend per one point = 0.042		*P* for trend per one point = 0.026		*P* for trend per one point = 0.220	

DBD item counts								
None	1.00		1.00		1.00		1.00	
One	1.35 (0.59–3.08)	0.481	0.82 (0.51–1.32)	0.424	1.29 (0.73–2.29)	0.384	0.78 (0.44–1.39)	0.406
Two to three	2.50 (1.23–5.12)	0.013	1.05 (0.65–1.68)	0.853	1.47 (0.86–2.51)	0.165	1.45 (0.86–2.43)	0.165
Four or more	2.16 (1.06–4.42)	0.036	1.64 (0.96–2.81)	0.072	1.55 (0.91–2.65)	0.107	1.52 (0.79–2.93)	0.212
	*P* for trend = 0.020		*P* for trend = 0.115		*P* for trend = 0.105		*P* for trend = 0.079	
	*P* for trend per one item = 0.015		*P* for trend per one item = 0.017		*P* for trend per one point = 0.012		*P* for trend per one item = 0.106	

***Females***								
DBD total score								
Q1 (lowest)	1.00		1.00		1.00		1.00	
Q2	1.16 (0.44–3.07)	0.770	1.48 (0.86–2.56)	0.161	1.08 (0.59–1.97)	0.805	2.05 (0.96–4.39)	0.073
Q3	1.81 (0.74–4.41)	0.196	1.25 (0.71–2.21)	0.443	0.98 (0.56–1.71)	0.944	2.80 (1.26–6.21)	0.015
Q4 (highest)	1.02 (0.43–2.42)	0.965	1.34 (0.71–2.54)	0.366	0.92 (0.52–1.63)	0.782	1.96 (0.77–5.02)	0.166
	*P* for trend = 0.715		*P* for trend = 0.523		*P* for trend = 0.630		*P* for trend = 0.116	
	*P* for trend per one point = 0.807		*P* for trend per one point = 0.334		*P* for trend per one point = 0.611		*P* for trend per one point = 0.597	

DBD item counts								
None	1.00		1.00		1.00		1.00	
One	0.98 (0.48–2.00)	0.961	0.82 (0.48–1.42)	0.486	0.87 (0.49–1.55)	0.633	0.80 (0.41–1.58)	0.524
Two to three	1.01 (0.55–1.86)	0.977	0.81 (0.49–1.35)	0.425	0.87 (0.53–1.43)	0.583	0.94 (0.49–1.78)	0.845
Four or more	0.98 (0.52–1.83)	0.947	1.20 (0.64–2.23)	0.569	1.09 (0.65–1.82)	0.748	1.04 (0.44–2.44)	0.930
	*P* for trend = 0.960		*P* for trend = 0.800		*P* for trend = 0.644		*P* for trend = 0.917	
	*P* for trend per one item = 0.857		*P* for trend per one item = 0.718		*P* for trend per one item = 0.558		*P* for trend per one item = 0.859	

BADL, basic activities of daily living; CI, confidence interval; DBD, Dementia Behavior Disturbance Scale; HR, hazard ratio.

Adjusted for age, living arrangement, education, economic status, Body Mass Index, basic activities of daily living (ADL), instrumental ADL, comorbidities, dementia types, antidementia drug use, antipsychotic drug use, and cognitive function.

^a^With difficulty in BADL: Barthel Index (BI) <100 points (*n* = 306–312 for males, 510–513 for females); without difficulty in BADL: BI = 100 points (*n* = 753–759 for males, 1,168–1,171 for females).

^b^With severely impaired cognitive function: Mini-Mental State Examination (MMSE) <21 points (*n* = 426–428 for males, 838–841 for females); with moderately/severely impaired cognitive function: MMSE ≥21 points (*n* = 637–639 for males, 840–843 for females).

Missing data were imputed using a multiple imputation approach.

Table 5 shows the association of each DBD item with mortality. In males, items of #3 “Lack of interest in daily activities,” #6 “Sleeps excessively during the day,” and #12 “Refuses to be helped with personal care” indicated a significantly higher mortality risk; in model 2, the HRs were 1.32 (95% CI, 1.01–1.73) for #3 (*P* = 0.044), 1.75 (95% CI, 1.35–2.27) for #6 (*P* < 0.001), and 1.53 (95% CI, 1.02–2.29) for #12 (*P* = 0.041). In the analysis in which DBD items were introduced simultaneously into the model, #6 remained associated with a higher risk of mortality (model 3, HR 1.69; 95% CI, 1.28–2.25, *P* < 0.001).

**Table 5.** Association between DBD items and mortality, based on multivariable Cox proportional hazards regression analysis

	Model 1 (crude model)		Model 2 (adjusted model)^a,b^		Model 3 (adjusted model)^a,c^	
		
	HR (95% CI)	*P*-value	HR (95% CI)	*P*-value	HR (95% CI)	*P*-value
***Males***						
DBD items						
#1 Asks same question repeatedly	1.61 (1.28–2.02)	<0.001	1.09 (0.85–1.40)	0.501	0.95 (0.72–1.25)	0.734
#2 Loses, misplaces, or hides things	1.57 (1.24–1.98)	<0.001	1.06 (0.82–1.37)	0.640	0.96 (0.72–1.29)	0.795
#3 Lack of interest in daily activities	1.87 (1.46–2.39)	<0.001	1.32 (1.01–1.73)	0.044	1.07 (0.79–1.44)	0.663
#4 Wakes up at night for no obvious reason	2.12 (1.48–3.03)	<0.001	1.26 (0.83–1.91)	0.278	1.06 (0.67–1.66)	0.813
#5 Makes unwarranted accusations	1.50 (0.96–2.34)	0.075	1.16 (0.73–1.86)	0.526	0.96 (0.57–1.63)	0.889
#6 Sleeps excessively during the day	2.52 (1.98–3.21)	<0.001	1.75 (1.35–2.27)	<0.001	1.69 (1.28–2.25)	<0.001
#8 Repeats the same action over and over	2.19 (1.54–3.12)	<0.001	1.47 (0.99–2.17)	0.055	1.29 (0.85–1.96)	0.237
#9 Is verbally abusive or curses	1.28 (0.82–1.99)	0.274	1.58 (0.98–2.53)	0.062	1.26 (0.74–2.14)	0.403
#12 Refuses to be helped with personal care	1.94 (1.34–2.81)	0.001	1.53 (1.02–2.29)	0.041	1.43 (0.90–2.27)	0.129
#13 Hoards things for no obvious reason	1.49 (1.03–2.18)	0.037	1.21 (0.80–1.84)	0.363	0.97 (0.62–1.52)	0.891
#19 Overeats	1.91 (1.35–2.70)	<0.001	1.45 (0.99–2.11)	0.055	1.13 (0.75–1.71)	0.547
#20 Is incontinent of urine	2.55 (1.78–3.64)	<0.001	1.22 (0.79–1.88)	0.381	1.11 (0.70–1.75)	0.659

***Females***						
DBD items						
#1 Asks same question repeatedly	1.18 (0.91–1.53)	0.203	0.87 (0.66–1.15)	0.340	0.91 (0.67–1.24)	0.553
#2 Loses, misplaces, or hides things	1.22 (0.95–1.56)	0.126	0.81 (0.62–1.06)	0.123	0.80 (0.59–1.08)	0.145
#3 Lack of interest in daily activities	1.48 (1.11–1.97)	0.008	1.03 (0.74–1.42)	0.879	1.00 (0.71–1.40)	0.978
#5 Makes unwarranted accusations	2.10 (1.37–3.21)	<0.001	1.49 (0.94–2.36)	0.092	1.75 (0.99–3.09)	0.058
#6 Sleeps excessively during the day	2.08 (1.54–2.81)	<0.001	1.33 (0.96–1.86)	0.089	1.35 (0.95–1.91)	0.091
#8 Repeats the same action over and over	1.57 (1.03–2.39)	0.037	0.84 (0.53–1.34)	0.473	0.77 (0.47–1.27)	0.312
#9 Is verbally abusive or curses	1.72 (1.10–2.69)	0.018	1.00 (0.62–1.62)	0.991	0.77 (0.43–1.39)	0.386
#12 Refuses to be helped with personal care	1.85 (1.18–2.89)	0.008	1.09 (0.67–1.77)	0.724	1.01 (0.59–1.75)	0.967
#13 Hoards things for no obvious reason	1.31 (0.94–1.84)	0.116	1.02 (0.71–1.47)	0.895	0.98 (0.66–1.47)	0.931
#19 Overeats	2.10 (1.41–3.13)	<0.001	1.50 (0.97–2.33)	0.072	1.53 (0.96–2.44)	0.078

CI, confidence interval; DBD, Dementia Behavior Disturbance Scale; HR, hazard ratio.

^a^Adjusted for age, living arrangement, education, economic status, Body Mass Index, basic activities of daily living (ADL), instrumental ADL, comorbidities, depressive symptoms, dementia types, antidementia drug use, antipsychotic drug use, and cognitive function.

^b^DBD items were separately introduced into the analytical model.

^c^DBD items were simultaneously introduced into the analytical model.

## DISCUSSION

To the best of our knowledge, this is the first large-scale study of BPSD and prognosis in people with cognitive impairment, including MCI. The results indicated that higher levels of BPSD, assessed using the DBD, were associated with an increased mortality risk among males, but not females. The associations were more pronounced in participants with BADL disabilities, and among the DBD items, lack of interest in daily activities, excessive daytime sleeping, and refusal to receive personal care showed a higher risk.

Many of the previous studies were conducted more than 15 years prior, with small sample sizes and inadequate adjustment for confounders.^15^^–^^25^ A recent large-scale study revealed a high mortality risk for moderate or worse BPSD, but the participants were from a nursing home-based registry and included people with relatively severe dementia^26^; there was insufficient evidence regarding mild cases. The present study showed that BPSD were associated with a higher mortality risk, even among participants with cognitive impairment, including MCI, and the robustness of the results was supported by several sensitivity analyses. Considering BPSD onset from the disease’s early stages, including MCI,^4^^,^^7^^–^^9^ the adverse prognostic impact of BPSD may need to be noted, even in individuals with more modest cognitive impairment. Although the nature of this observational study design could not determine causality, our findings provide additional evidence about the potential risk of BPSD as a factor affecting the prognosis of people with cognitive impairment.

The severity of BPSD often correlates with the degree of ADL and cognitive impairment.^4^ The association of BPSD with poor prognosis may only represent a surrogate association for dysfunctions. However, our stratified analysis showed that the higher mortality risk of BPSD in males was dose-responsively increased, regardless of ADL and cognitive impairment, suggesting a plausible association between BPSD severity and early death. Furthermore, the analyses found that the association was more marked in those with BADL dysfunctions. BPSD in those with daily living impairment may lead to more difficulty in caregiving, increased psychiatric drug use, and early admission, which may be associated with poor prognosis. Alternatively, behavioral problems combined with BADL impairments may increase the accident risk, such as falls and fractures, which can result in a poor prognosis. Because no statistical significance was found for the interaction testing, this study could not provide definitive conclusions on this synergistic effect, but clinicians and future research may need to focus on ADL functions in participants with BPSD.

We explored specific symptoms related to poor prognosis in BPSD, indicating that lack of interest in daily activities, excessive daytime sleeping, and refusal to receive personal care showed a higher mortality risk. The DBD used in this study mainly assesses behavioral symptoms and does not explicitly assess psychological symptoms, such as delusions and hallucinations, so the tool may fail to capture the full range of BPSD. Nonetheless, the results may be reasonable. Prior research has shown that apathy, along with hallucinations and agitation, among BPSD, increases mortality risk.^26^^,^^47^ The lack of interest in daily activities is considered a symptom related to apathy, which is partly consistent with prior research. Excessive daytime sleeping had a strong mortality risk, even with all DBD items simultaneously included in the analytical model; this common symptom of dementia, due to an impaired circadian rhythm,^4^ might be a proxy for severe cognitive decline or a later-stage sign of dementia.^48^^,^^49^ Alternatively, deficits in spontaneity lead to a rapid decline in the physical and cognitive function of people with cognitive impairment, which may be related to early death risk. Refusal to receive care is linked to psychotropic drug use, inappropriate care, and early institutionalization,^50^ potentially increasing mortality risk.

These specific symptoms associated with poor prognosis may partially explain the sex differences in the higher mortality risk in BPSD. Particularly, the frequencies of symptoms of lack of interest in daily activities and excessive daytime sleeping were found to be higher among males than females. A meta-analysis of sex differences in psychiatric symptoms showed that apathy is more severe in males than females among individuals with AD^31^; this finding is supported by another study.^34^ While the evidence is unclear for sex differences in the symptoms of excessive daytime sleeping in people with dementia, some studies suggest that apathy and sleep problems often appear together.^51^^,^^52^ These imply that apathy-related symptoms may affect prognosis and sex-related differences in people with cognitive impairment. Alternatively, the more prevalent symptoms of refusal of care in males might have prevented them from seeking medical care and treating comorbidities, affecting their poor prognosis. Since this study did not fully clarify the mechanisms and the causes of sex differences, further investigations are needed to identify the BPSD types related to poor prognosis and to elucidate the factors contributing to the sex differences.

This study’s strength is its large sample size and long-term follow-up, which enabled a variety of stratification and sensitivity analyses. Another is the enrollment of the participants from the memory clinic outpatients, facilitating a wide range of dementia types, including MCI, based on clinical diagnosis. However, this study also has several limitations. First, the collection of death information via mail survey may have systematically resulted in a loss to follow-up, especially in those who were males, lived alone, had dementia diagnosis, and cognitive and ADL impairment, potentially leading to selection bias.^36^ Although these factors were considered in the analytical model, we should be cautious about the potential overestimation of the results, including the effect of unmeasured factors on loss to follow-up. Further investigations with higher follow-up, such as using linkage to public registry data on death information, are needed. Second, the DBD does not evaluate some psychological symptoms, such as delusions and hallucinations, within BPSD. It may fail to provide a comprehensive BPSD assessment. Third, while BPSD can vary over time, this study was limited by single-time-point assessment at the first medical visit. Evaluating BPSD multiple times is necessary to examine their prognostic impact more accurately. Fourth, this study failed to identify cause-specific deaths and investigate the mechanisms behind the high mortality risk of BPSD. Fifth, in this study, people with all types of dementia were collectively analyzed; however, the association between BPSD and mortality may differ for each type of dementia. Because this study’s sample size was relatively small to analyze each type of dementia, further investigations with a larger sample size are required. Finally, the study results were based on participants from a single center and have limited generalizability. Future research involving multicenter recruitment is warranted.

In conclusion, this memory clinic-based longitudinal study of people with cognitive impairment indicated that higher levels of BPSD were associated with increased mortality risk among males. Our findings suggest that BPSD may be a potential factor related to poor prognosis, underscoring the importance of addressing BPSD from the early stages of cognitive impairment.

## SUPPLEMENTARY MATERIAL

eTable 1. Summary statistics for DBD scores and item counts

eTable 2. Association between DBD and mortality, based on multivariable Cox proportional hazards regression analysis with complete case analysis

eTable 3. Sensitivity analyses excluding event participants at 6, 12, 18, and 24 months of follow-up on association between DBD and mortality, based on multivariable Cox proportional hazards regression analysis

eTable 4. Sensitivity analyses by restricting or excluding participants with each dementia diagnosis on association between DBD and mortality, based on multivariable Cox proportional hazards regression analysis

eTable 5. Interaction testing by BADL and cognitive function on the association between DBD and mortality, based on multivariable Cox proportional hazards regression analysis

eFigure 1. Histograms of the DBD scores and item counts. DBD, Dementia Behavior Disturbance Scale. Panels **A** and **B** for DBD scores and item counts, respectively.

eFigure 2. Complementary log-log survival plots. DBD, Dementia Behavior Disturbance Scale. Panel **A** for males based on the DBD scores, **B** for males based on the DBD item counts, **C** for females on the DBD scores, and **D** for females based on the DBD item counts. The results of testing the assumption of proportional hazards based on the Schoenfeld residuals were follows: *P* = 0.076 (**A**), 0.136 (**B**), 0.350 (**C**), and 0.776 (**D**).
