# Supplementary material for: Behavioral and Psychological Symptoms of Dementia and Mortality Risk Among People With Cognitive Impairment: An 8-year Longitudinal Study From the NCGG-STORIES
Source: J Epidemiol. 2024 Nov 5;34(11):543–52. doi: 10.2188/jea.JE20230343 (PMC11464853; doi:10.2188/jea.JE20230343)
Supplement: Supplementary file 1 [file je-34-543-s001.pdf]

eTable 1. Summary statistics for DBD scores and item counts

	Mean	SD	Median	IQR	Min	Max
DBD scores						
Males	14.8	11.5	12	13	0	70
Females	15.2	11.6	12	13	0	83
DBD item counts						
Males	1.9	2.4	1	3	0	16
Females	2.1	2.4	2	3	0	20

DBD, Dementia Behavior Disturbance Scale; IQR, interquartile range; SD, standard deviation.

eTable 2. Association between DBD and mortality, based on multivariable Cox proportional hazards regression analysis with complete case analysis

	Males (n=969)				Females (n=1,485)			
	Model 1 (crude model)		Model 2 (adjusted model)[a]		Model 1 (crude model)		Model 2 (adjusted model)[a]	
	HR (95% CI)	*P*-value	HR (95% CI)	*P*-value	HR (95% CI)	*P*-value	HR (95% CI)	*P*-value
DBD total score								
Q1 (lowest)	1.00		1.00		1.00		1.00	
Q2	1.11 (0.73–1.50)	0.589	1.08 (0.67–1.49)	0.724	1.71 (1.27–2.15)	0.017	1.44 (0.95–1.93)	0.146
Q3	1.68 (1.33–2.03)	0.004	1.21 (0.82–1.60)	0.332	2.24 (1.82–2.65)	< 0.001	1.56 (1.07–2.05)	0.076
Q4 (highest)	3.05 (2.73–3.38)	< 0.001	1.48 (1.08–1.87)	0.051	2.84 (2.44–3.24)	< 0.001	1.26 (0.74–1.79)	0.386
	P for trend<0.001		*P* for trend=0.043		*P* for trend<0.001		*P* for trend=0.511	
	P for trend per one point<0.001		*P* for trend per one point=0.015		*P* for trend per one point<0.001		*P* for trend per one point=0.343	
DBD item counts								
None	1.00		1.00		1.00		1.00	
One	1.15 (0.78–1.52)	0.454	0.95 (0.55–1.36)	0.820	1.07 (0.65–1.49)	0.743	0.80 (0.34–1.27)	0.355
Two to three	2.05 (1.73–2.38)	< 0.001	1.36 (1.00–1.73)	0.098	1.40 (1.03–1.76)	0.070	0.94 (0.53–1.35)	0.775
Four or more	3.17 (2.85–3.50)	< 0.001	1.44 (1.05–1.84)	0.070	2.20 (1.83–2.58)	< 0.001	1.10 (0.63–1.57)	0.700
	P for trend<0.001		*P* for trend=0.024		*P* for trend<0.001		*P* for trend=0.580	
	P for trend per one item<0.001		*P* for trend per one item=0.004		*P* for trend per one item<0.001		*P* for trend per one item=0.397	

CI, confidence interval; DBD, Dementia Behavior Disturbance Scale; HR, hazard ratio.

[a]Adjusted for age, living arrangement, education, economic status, Body Mass Index, basic activities of daily living (ADL), instrumental ADL, comorbidities, depressive symptoms, dementia types, antidementia drug use, psychoactive drug use, and cognitive function.

eTable 3. Sensitivity analyses excluding event participants at 6, 12, 18, and 24 months of follow-up on association between DBD and mortality, based on multivariable Cox proportional hazards regression analysis

	Model 3 (excluding event participants within a 6-month period)		Model 4 (excluding event participants within a 12-month period)		Model 5 (excluding event participants within an 18-month period)		Model 6 (excluding event participants within a 24-month period)	
	HR (95% CI)	*P*-value	HR (95% CI)	*P*-value	HR (95% CI)	*P*-value	HR (95% CI)	*P*-value
Males								
DBD total score								
Q1 (lowest)	1.00		1.00		1.00		1.00	
Q2	1.05 (0.69–1.58)	0.834	0.96 (0.63–1.48)	0.862	0.98 (0.63–1.54)	0.933	0.93 (0.58–1.49)	0.752
Q3	1.37 (0.94–1.99)	0.103	1.26 (0.86–1.87)	0.238	1.34 (0.90–2.01)	0.154	1.27 (0.83–1.94)	0.269
Q4 (highest)	1.60 (1.10–2.33)	0.015	1.58 (1.07–2.31)	0.021	1.62 (1.08–2.42)	0.021	1.44 (0.94–2.21)	0.099
	P for trend=0.007		*P* for trend=0.011		*P* for trend=0.010		*P* for trend=0.056	
	P for trend per one point=0.004		*P* for trend per one point=0.003		*P* for trend per one point=0.002		*P* for trend per one point=0.032	
DBD item counts								
None	1.00		1.00		1.00			
One	0.93 (0.62–1.38)	0.707	0.85 (0.56–1.28)	0.432	0.86 (0.56–1.33)	0.512	0.87 (0.55–1.37)	0.553
Two to three	1.49 (1.04–2.12)	0.030	1.46 (1.01–2.11)	0.046	1.50 (1.02–2.21)	0.039	1.41 (0.94–2.13)	0.101
Four or more	1.50 (1.02–2.22)	0.041	1.56 (1.04–2.33)	0.033	1.59 (1.04–2.42)	0.033	1.51 (0.97–2.37)	0.073
	P for trend=0.008		*P* for trend=0.005		*P* for trend=0.006		*P* for trend=0.022	
	P for trend per one item=0.003		*P* for trend per one item<0.001		*P* for trend per one item<0.001		*P* for trend per one item=0.008	
Females								
DBD total score								
Q1 (lowest)	1.00		1.00		1.00		1.00	

Q2	1.24 (0.78–1.97)	0.354	1.18 (0.74–1.89)	0.494	1.00 (0.62–1.63)	0.994	0.96 (0.57–1.59)	0.863
Q3	1.27 (0.81–1.99)	0.305	1.21 (0.77–1.91)	0.413	1.00 (0.63–1.61)	0.985	0.99 (0.61–1.62)	0.969
Q4 (highest)	1.02 (0.63–1.65)	0.923	1.03 (0.63–1.68)	0.899	0.91 (0.55–1.50)	0.715	0.92 (0.54–1.56)	0.757
	P for trend=0.880		*P* for trend=0.981		*P* for trend=0.691		*P* for trend=0.791	
	P for trend per one point=0.599		*P* for trend per one point=0.408		*P* for trend per one point=0.600		*P* for trend per one point=0.496	
DBD item counts								
None	1.00		1.00		1.00		1.00	
One	0.85 (0.56–1.30)	0.457	0.90 (0.58–1.40)	0.646	0.81 (0.51–1.29)	0.385	0.77 (0.48–1.24)	0.288
Two to three	0.86 (0.58–1.26)	0.432	0.92 (0.62–1.37)	0.687	0.84 (0.56–1.27)	0.410	0.77 (0.51–1.19)	0.241
Four or more	0.96 (0.63–1.46)	0.839	1.02 (0.66–1.59)	0.921	0.94 (0.59–1.49)	0.785	0.89 (0.55–1.44)	0.642
	P for trend=0.879		*P* for trend=0.881		*P* for trend=0.849		*P* for trend=0.672	
	P for trend per one item=0.835		*P* for trend per one item=0.682		*P* for trend per one item=0.898		*P* for trend per one item=0.910	

CI, confidence interval; DBD, Dementia Behavior Disturbance Scale; HR, hazard ratio.

Adjusted for age, living arrangement, education, economic status, Body Mass Index, basic activities of daily living (ADL), instrumental ADL, comorbidities, depressive symptoms, dementia types, antidementia drug use, psychoactive drug use, and cognitive function.

Missing data were imputed by a multiple imputation approach.

Model 3: excluding 17 participants for males and 4 participants for females; Model 4: excluding 41 participants for males and 17 participants for females; Model 5: excluding 61 participants for males and 40 participants for females; Model 6: excluding 90 participants for males and 55 participants for females.

eTable 4. Sensitivity analyses by restricting or excluding participants with each dementia diagnosis on association between DBD and mortality, based on multivariable Cox proportional hazards regression analysis

	Restricted to participants with AD (n=520 for males; 1,067 for females)		Excluding participants with MCI (n=669 for males; 1,212 for females)		Excluding participants with AD (n=545 for males; 614 for females)		Excluding participants with DLB (n=991 for males; 1,582 for females)		Excluding participants with FLD (n=1041 for males; 1,660 for females)		Excluding participants with VAD (n=1014 for males; 1,656 for females)	
	HR (95% CI)	*P*-value	HR (95% CI)	*P*-value	HR (95% CI)	*P*-value	HR (95% CI)	*P*-value	HR (95% CI)	*P*-value	HR (95% CI)	*P*-value
Males												
DBD total score												
Q1 (lowest)	1.00		1.00		1.00		1.00		1.00		1.00	
Q2	1.20 (0.69–2.10)	0.523	1.24 (0.77–2.02)	0.380	1.03 (0.55–1.91)	0.937	1.10 (0.73–1.67)	0.647	1.00 (0.67–1.51)	0.984	1.03 (0.68–1.57)	0.872
Q3	1.49 (0.90–2.47)	0.127	1.43 (0.93–2.21)	0.108	1.37 (0.74–2.55)	0.317	1.23 (0.83–1.83)	0.297	1.26 (0.87–1.82)	0.232	1.31 (0.90–1.93)	0.163
Q4 (highest)	1.92 (1.16–3.17)	0.012	1.84 (1.20–2.82)	0.006	1.46 (0.79–2.68)	0.229	1.63 (1.11–2.41)	0.014	1.53 (1.06–2.20)	0.025	1.61 (1.10–2.35)	0.016
	P for trend=0.007		*P* for trend=0.004		*P* for trend=0.181		*P* for trend=0.012		*P* for trend=0.015		*P* for trend=0.008	
	P for trend per one point=0.039		*P* for trend per one point=0.003		*P* for trend per one point=0.143		*P* for trend per one point=0.051		*P* for trend per one point=0.009		*P* for trend per one point=0.003	
DBD item counts												
None	1.00		1.00		1.00		1.00		1.00		1.00	
One	0.92 (0.54–1.57)	0.764	1.07 (0.69–1.68)	0.754	1.13 (0.62–2.04)	0.692	0.93 (0.62–1.39)	0.715	0.98 (0.66–1.46)	0.932	0.90 (0.60–1.35)	0.619
Two to three	1.73 (1.08–2.78)	0.024	1.56 (1.04–2.34)	0.033	1.48 (0.82–2.65)	0.194	1.56 (1.07–2.26)	0.021	1.50 (1.06–2.14)	0.025	1.41 (0.98–2.02)	0.067
Four or more	1.79 (1.10–2.94)	0.022	1.65 (1.08–2.51)	0.020	1.36 (0.69–2.69)	0.379	1.55 (1.04–2.31)	0.033	1.53 (1.04–2.25)	0.032	1.61 (1.08–2.40)	0.019
	P for trend=0.004		*P* for trend=0.006		*P* for trend=0.236		*P* for trend=0.006		*P* for trend=0.007		*P* for trend=0.004	
	P for trend per one item=0.008		*P* for trend per one item=0.002		*P* for trend per one item=0.442		*P* for trend per one item=0.021		*P* for trend per one item=0.002		*P* for trend per one item<0.001	
Females												
DBD total score												

Q1 (lowest)	1.00		1.00		1.00		1.00		1.00		1.00	
Q2	0.94 (0.55–1.59)	0.806	1.09 (0.66–1.81)	0.725	2.73 (1.04–7.18)	0.054	1.10 (0.69–1.76)	0.691	1.26 (0.79–2.01)	0.338	1.24 (0.78–1.98)	0.356
Q3	0.95 (0.58–1.57)	0.847	1.10 (0.68–1.77)	0.704	4.26 (1.59–11.41)	0.008	1.26 (0.80–1.99)	0.319	1.33 (0.85–2.09)	0.216	1.31 (0.83–2.06)	0.246
Q4 (highest)	0.84 (0.49–1.42)	0.508	0.85 (0.52–1.40)	0.522	2.07 (0.70–6.11)	0.200	1.05 (0.65–1.70)	0.841	1.10 (0.68–1.78)	0.697	1.07 (0.66–1.74)	0.789
	P for trend=0.516		*P* for trend=0.381		*P* for trend=0.192		*P* for trend=0.805		*P* for trend=0.846		*P* for trend=0.944	
	P for trend per one point=0.624		*P* for trend per one point=0.908		*P* for trend per one point=0.524		*P* for trend per one point=0.419		*P* for trend per one point=0.634		*P* for trend per one point=0.473	
DBD item counts												
None	1.00		1.00		1.00		1.00		1.00		1.00	
One	0.78 (0.47–1.30)	0.347	0.71 (0.45–1.13)	0.155	1.00 (0.43–2.34)	0.994	0.94 (0.60–1.47)	0.786	0.88 (0.57–1.35)	0.559	0.85 (0.55–1.31)	0.464
Two to three	0.79 (0.50–1.24)	0.302	0.75 (0.51–1.13)	0.172	1.24 (0.57–2.70)	0.600	0.97 (0.64–1.46)	0.882	0.90 (0.61–1.33)	0.594	0.89 (0.60–1.32)	0.564
Four or more	0.88 (0.53–1.44)	0.605	0.81 (0.52–1.25)	0.344	2.02 (0.81–5.03)	0.143	1.16 (0.73–1.83)	0.527	1.04 (0.68–1.59)	0.874	0.99 (0.65–1.53)	0.973
	P for trend=0.693		P for trend=0.471		*P* for trend=0.148		*P* for trend=0.517		*P* for trend=0.828		*P* for trend=0.930	
	P for trend per one item=0.963		*P* for trend per one item=0.917		*P* for trend per one item=0.210		*P* for trend per one item=0.594		*P* for trend per one item=0.918		*P* for trend per one item=0.729	

AD, Alzheimer’s dementia; CI, confidence interval; DBD, Dementia Behavior Disturbance Scale; DLB, dementia with Lewy bodies; FTLD, frontotemporal lobar degeneration; HR, hazard ratio; VAD, vascular dementia.

Adjusted for age, living arrangement, education, economic status, Body Mass Index, basic activities of daily living (ADL), instrumental ADL, comorbidities, depressive symptoms, dementia types, antidementia drug use, psychoactive drug use, and cognitive function.

Missing data were imputed by a multiple imputation approach.

eTable 5. Interaction testing by BADL and cognitive function on the association between DBD and mortality, based on multivariable Cox proportional hazards regression analysis

	Males		Females	
	HR (95% CI)	*P*-value	HR (95% CI)	*P*-value
DBD total score × BADL (without difficulty=0, with difficulty=1)				
Q1 (lowest)	Ref		Ref	
Q2	1.69 (0.59–4.80)	0.326	1.03 (0.35–3.03)	0.952
Q3	2.36 (0.91–6.14)	0.079	1.51 (0.54–4.21)	0.432
Q4 (highest)	1.64 (0.64–4.23)	0.308	1.11 (0.40–3.09)	0.838
Per quartile point	1.12 (0.87–1.44)	0.381	0.99 (0.76–1.29)	0.928
Per one point	1.00 (0.98–1.02)	0.847	1.00 (0.97–1.02)	0.927
DBD item counts× BADL (without difficulty=0, with difficulty=1)				
None	Ref		Ref	
One	1.99 (0.82–4.83)	0.132	1.09 (0.46–2.61)	0.839
Two to three	2.47 (1.11–5.53)	0.028	1.11 (0.51–2.41)	0.787
Four or more	1.80 (0.79–4.11)	0.164	0.81 (0.35–1.84)	0.608
Per group of item count	1.17 (0.92–1.48)	0.204	0.97 (0.75–1.25)	0.802
Per one item count	0.97 (0.87–1.07)	0.504	0.99 (0.88–1.12)	0.904
DBD total score × cognitive function (with mildly impairment=0, with moderately/severely impairment=1)				
Q1 (lowest)	Ref		Ref	
Q2	1.36 (0.6–3.08)	0.459	1.03 (0.35–3.03)	0.952
Q3	0.92 (0.44–1.93)	0.833	1.51 (0.54–4.21)	0.432
Q4 (highest)	1.43 (0.68–2.98)	0.348	1.11 (0.40–3.09)	0.838
Per quartile point	1.08 (0.86–1.36)	0.516	0.77 (0.59–0.99)	0.046
Per one point	1.01 (0.98–1.03)	0.591	0.99 (0.96–1.01)	0.234
DBD item counts × cognitive function (with mildly impairment=0, with moderately/severely impairment=1)				
None	Ref		Ref	
One	1.44 (0.65–3.18)	0.373	1.02 (0.43–2.42)	0.961
Two to three	1.06 (0.52–2.14)	0.877	0.84 (0.39–1.83)	0.670
Four or more	1.14 (0.52–2.49)	0.739	0.83 (0.34–2.00)	0.672
Per group of item count	1.01 (0.80–1.28)	0.935	0.94 (0.71–1.24)	0.674
Per one item count	1.01 (0.91–1.12)	0.829	0.97 (0.85–1.09)	0.572

BADL, basic activity of daily living; CI, confidence interval; DBD, Dementia Behavior Disturbance Scale; HR, hazard ratio.

Adjusted for age, living arrangement, education, economic status, Body Mass Index, BADL, instrumental ADL, comorbidities, depressive symptoms, dementia types, antidementia drug use, psychoactive drug use, and cognitive function.

Missing data were imputed by a multiple imputation approach.

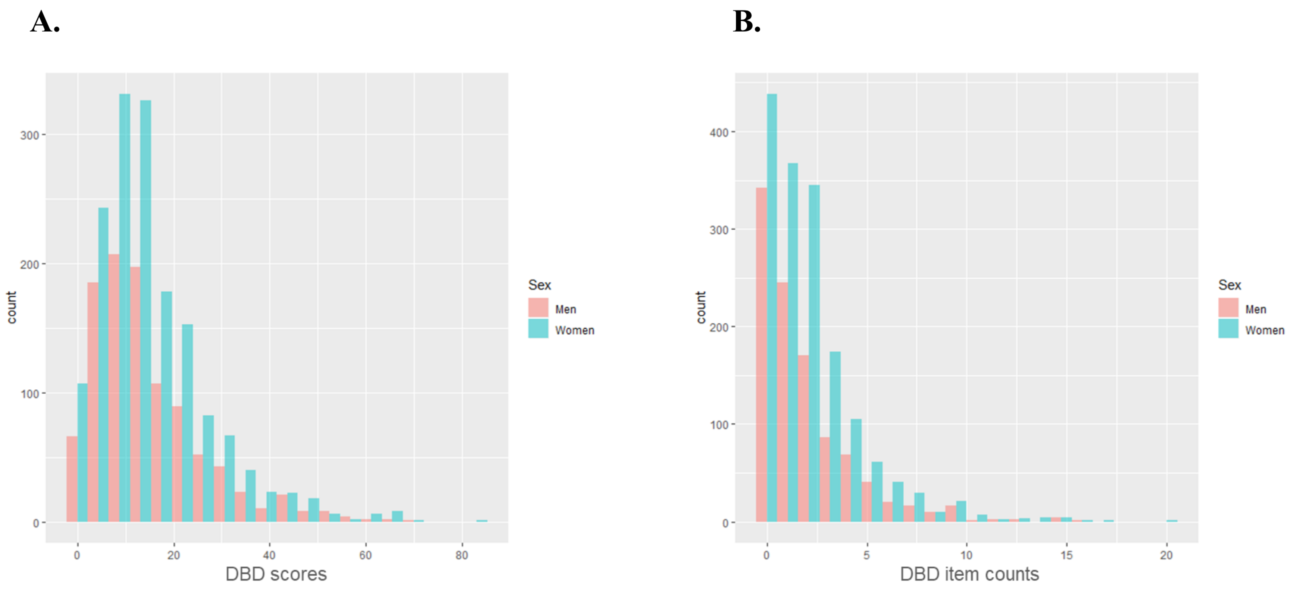

eFigure 1. Histograms of the DBD scores and item counts. DBD, Dementia Behavior Disturbance Scale. Panels **A** and **B** for DBD scores and item counts, respectively.

A.

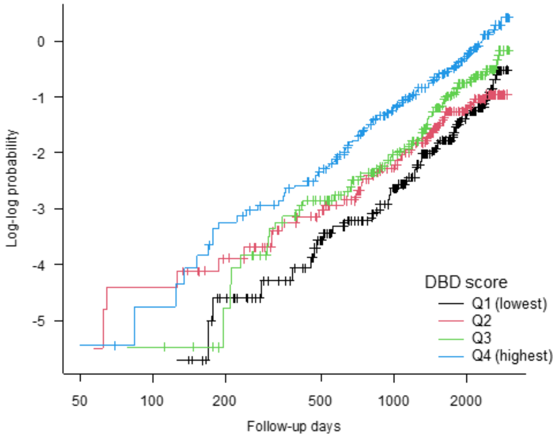

B.

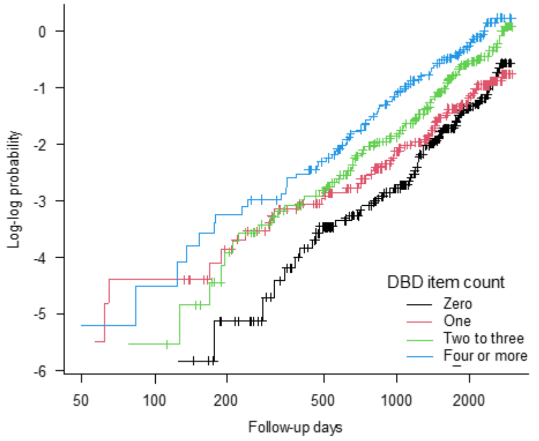

C.

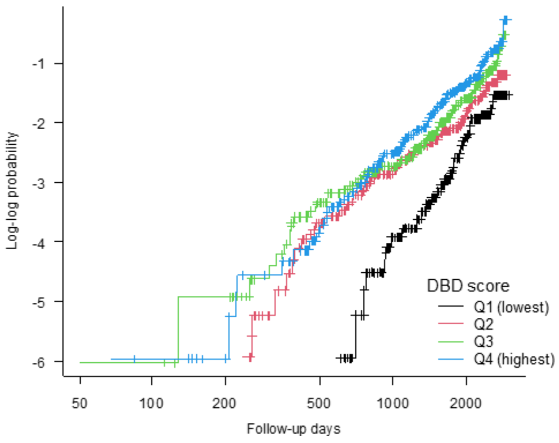

D.

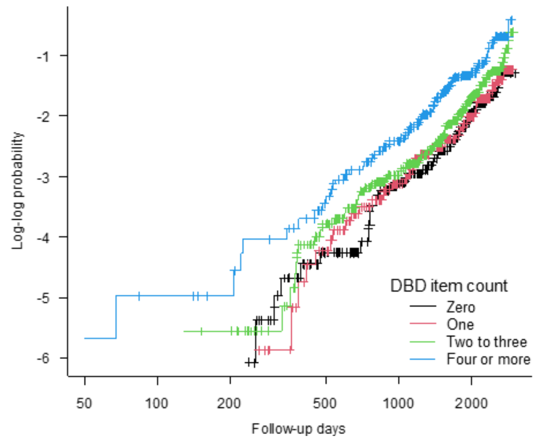

eFigure 2. Complementary log-log survival plots. DBD, Dementia Behavior Disturbance Scale. Panel **A** for males based on the DBD scores, **B** for males based on the DBD item counts, **C** for females on the DBD scores, and **D** for females based on the DBD item counts. The results of testing the assumption of proportional hazards based on the Schoenfeld residuals were follows: P=0.076 (**A**), 0.136 (**B**), 0.350 (**C**), and 0.776 (**D**).
